# Liver Parenchymal Transection Through Radiofrequency Ablation Using a Radial Probe: Technical Report of a New Modality

**DOI:** 10.7759/cureus.20130

**Published:** 2021-12-03

**Authors:** Venu Bhargava Mulpuri, Prasanth Gurijala, Bhaskar R Yerolla, Gokul Kumar, Ashwini Dutt

**Affiliations:** 1 Department of Surgical Gastroenterology, Employee State Insurance Corporation Medical College and Hospital, Hyderabad, IND; 2 Department of General Surgery, Employee State Insurance Corporation Medical College, Hyderabad, IND; 3 Department of General Surgery, Employee State Insurance Corporation Hospital, Hyderabad, IND; 4 Department of General Surgery, Employee State Insurance Corporation Medical College and Hospital, Hyderabad, IND

**Keywords:** radiofrequency ablation (rfa), liver parenchymal transection with minimal blood loss, newer technique of liver parenchymal transection, liver transection using radial probe, liver parenchymal transection using radiofrequency ablation, liver parenchymal transection

## Abstract

Various techniques have been reported to reduce blood loss during a parenchymal transection, and the radiofrequency ablation (RFA) technique is one of them. Owing to the charring of the adjacent liver tissue and the inability to use the conventional RFA techniques near major vessels, this study used a radial fiber of RFA. This technical report thus describes a technique to perform parenchymal transection using a radial fiber as well as its advantages and disadvantages. A radial fiber dissipates the energy radially and it has the added advantage of placing along the same and perpendicular axis of the liver parenchyma; it has been used in three patients in this study. The total intraoperative blood loss was 30-50 ml during parenchymal transection, and the intraoperative duration was 120-170 min. Bile leak was noted in one patient, which was then managed using the conventional treatment. Through the present technique, the fiber can be used in the vicinity of major blood vessels and necrosis and charring can be prevented. Although radial fiber has some advantages, it remains in the preliminary stage and requires further validation.

## Introduction

Surgical resection is the treatment of choice for primary and metastatic liver tumors [1]. The major concern following the surgery is post hepatectomy liver failure [2,3]. Intraoperative blood loss is one of the factors responsible for post hepatectomy liver failure [4]. Several studies have shown that intraoperative blood loss is associated with higher mortality and morbidity as well as longer hospital stay [4]. Various techniques have been developed to reduce intraoperative blood loss during liver tumor resection, including hypotensive anesthesia, the Pringle maneuver, or total vascular exclusion [2]. Parenchymal transection can be performed using the finger fracture technique, Kelly crush technique, ultrasonic dissector, water-jet, or laserjet [3]. Pai et al. [4] first described the use of radiofrequency ablation (RFA) in parenchymal transection; they used a single-probe RFA to reduce intraoperative blood loss. They used a metal probe that has to be inserted perpendicularly into the liver parenchyma, whereas in the technique which we describe probe can be placed horizontally and perpendicular to the liver parenchyma [5]. Another technique used for this purpose is the inline RFA technique, where a precise RFA area is created between the electrodes of alternating polarity [6-8]. However, the primary limitation of these techniques is the charring of the adjacent liver tissue and the need for the identification of the major vessels before placing the probes [9]. Therefore, this study describes a new technique to avoid the charring of the liver tissue and any injury to major biliovascular structures through the use of a radial RFA probe for the ablation of varicose veins.

## Technical report

**Figure 1:**
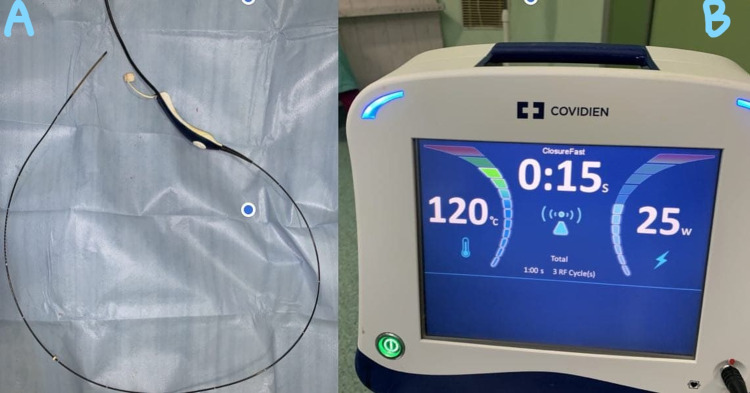
A: Radial probe of the RFA B: Monitor showing the temperature achieved during the procedure RFA: radiofrequency ablation

**Figure 2:**
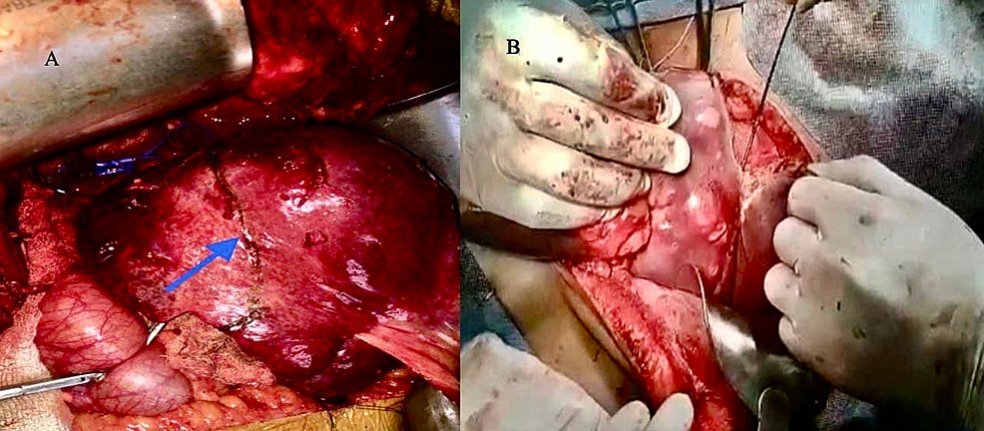
A: Parenchymal transection line, B: image showing insertion of RFA for parenchymal transection RFA: radiofrequency ablation

**Figure 3:**
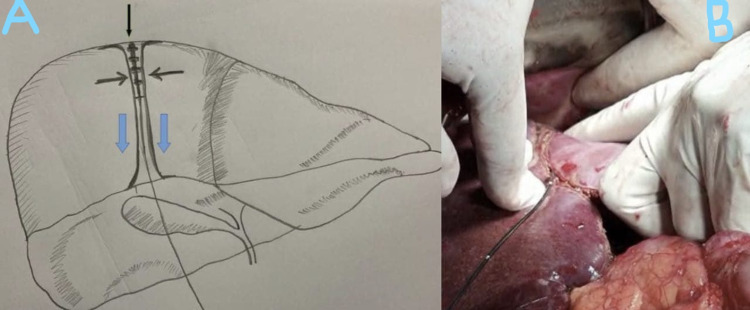
A: Representative diagram showing how to introduce RFA probe into the liver parenchyma, B: RFA probe is introduced in to the liver parenchyma along the same axis of the transection plane RFA: radiofrequency ablation

**Figure 4:**
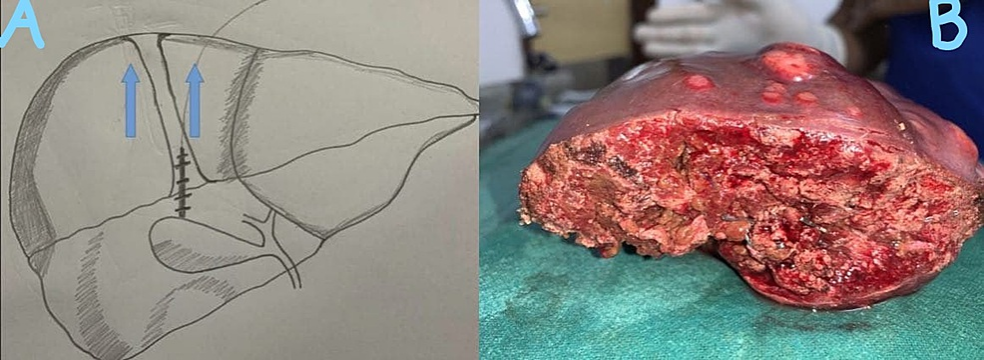
A: Representative diagram showing RFA probe being introduced into the liver parenchyma along the axis perpendicular to the transection plane, B: Transected liver without any charring at the cut margin RFA: radiofrequency ablation

Under general anesthesia, after identifying and controlling the vascular supply of the segment that has to be divided, the transection plane is marked using a monopolar coagulation device. Pringle maneuver has been done only in one patient and two other patients did not tolerate the maneuver. Once the transection plane is marked, an initial 2-cm transection is performed using monopolar and bipolar coagulation devices so it has to have enough liver parenchyma around the radial probe of RFA. The fast-closure RFA system from Covidien (Dublin, Ireland) was used in this study after obtaining consent from the patients and ethical clearance from the institute. The system used was a 60-cm length fiber with a 7-cm long heating element; the diameter of the fiber was 2.3 mm. The maximum power setting used was 40 W, and the default maximum temperature was 120°C. The radial probe of RFA (Figure 1) is placed horizontally along the line of transection as shown in Figure 2, and the two edges of the liver are approximated with fingers; ablation is performed for 20 seconds with the temperature rising to 120°C (Figure 2). Next, the catheter is withdrawn and other areas along the transection line are ablated similarly as the radial RFA probe heats only 7 cm. Just below the transection line, an RFA probe is introduced into the liver parenchyma along the same axis of the transection plane, and ablation is performed as described (Figure 3). After ablation, the tissue can be transected using a Kelly clamp, leaving behind the biliovascular structures, and either suture-ligated or divided after clip application. No charring occurs along the transection line, and any injury to the major vascular structures can be avoided. The RFA probe can also be placed perpendicular to the transection line after performing intraoperative ultrasound and was ablated similarly (Figure 4). During the final part of parenchymal transection to avoid injury to inferior vena cava (IVC), hanging maneuver was performed. To date, we have followed this technique in three patients (two patients underwent segmental resections and one patient underwent left hemihepatectomy); the blood loss was 30-50 ml during the parenchymal transection. Postoperative bile leak was observed in one patient, which was managed conventionally.

## Discussion

For liver tumors, RFA ablation aims to destroy the tumor tissue by transferring thermal energy to the tissue. Weber et al. [5] introduced a technique for precoagulation of the liver parenchyma using radiofrequency energy, creating an ischemic zone before transection of the liver parenchyma. Precoagulation is an important technique used to reduce intraoperative blood loss, particularly in patients with cirrhosis.

Several studies have used RFA to aid liver resection and reduce the amount of blood loss during the parenchymal transection [4,8,10]. To attain this objective, either a single- or multiprobe RFA technique can be used [11]. With this interesting technique, radiofrequency energy is applied to healthy liver tissue. The heat produced seals the major biliary and blood vessels, enabling the resection of liver parenchyma without excessive blood loss [12].

Parenchymal transection is the treatment of choice for patients with liver tumors. In the comparison of the crush-clamp technique and RFA, intraoperative blood loss was lower in the latter [13,14] but infection-related complications were lower in the former [14]. This might be because of excessive charring or coagulation due to heat energy. The crush-clamp technique is also the most cost-effective one. However, this technique requires a Pringle maneuver. Once the blood vessels and bile ducts are exposed, they must be divided using sutures, bipolar electrocautery, vessel-sealing devices, or vascular clips [15].

RFA is a robust ablation method, the experience with different power generators and ablation catheters raised the question of practical differences between different combinations. RFA cannot be used close to the hilum or vena cava, and a necrotic resection margin of 1 cm is left on the remnant liver with a relatively increased risk of bile leak and abscess formation [15,16]. To address this problem, a radial RFA fiber for ablating varicose veins was used in this study. With this fiber, heat energy is transmitted radially, coagulating small blood vessels and bile radicles. However, this technique did not coagulate the larger blood vessels such as the middle hepatic vein, segmental veins, and segmental sheaths. While using this technique, surgeons must ensure that the entire active end of the probe is covered with liver tissue for better dissipation of the heat energy. Devices with a larger distance between the electrodes like in inline RFA tend to leave a gap in the ablation area at lower power settings, which may be prevented by multiple ablations in the axis of the probe.

The conventional RFA technique described by Pai et al. [4] used a probe with a 3 cm active electrode, a thermocouple on the tip to monitor temperature and impedance, and coaxial cannulae through which chilled saline is circulated to prevent tissue boiling and cavitation. The radial fiber RFA which we used had a 7 cm active electrode thereby decreasing the number of applications. The radial fiber RFA doesn’t require cooling with chilled saline and it did not result in cavitation. This technique has the following advantages: a) it can be used in the vicinity of the major blood vessels and bile ducts without causing damage to major blood vessels, b) necrotic resection margin can be avoided, and c) blood loss can be reduced. However, the two main disadvantages associated with the use of this technique are the need for identifying major segmental vessels, which require either clip placement or suture ligation and division, and the longer surgery duration than the conventional RFA techniques. The major limitation of this new technique is that it is not validated yet and it requires further exploration in many patients before being prescribed for general use.

## Conclusions

RFA assisted liver parenchymal transection reduces blood loss. The new technique using a radial probe described in this report is easily reproducible. In addition, it has added advantages compared with previously described techniques as it decreases the charring of the surrounding liver parenchyma. However, this technique is still in the preliminary stage and requires further validation in many patients before being prescribed for general use.

## References

[1] Imai D, Maeda T, Wang H (2021). Risk factors for and outcomes of intraoperative blood loss in liver resection for hepatocellular tumors. Am Surg.

[2] Qian NS, Liao YH, Cai SW, Raut V, Dong JH (2013). Comprehensive application of modern technologies in precise liver resection. Hepatobiliary Pancreat Dis Int.

[3] Gurusamy KS, Pamecha V, Sharma D, Davidson BR (2009). Techniques for liver parenchymal transection in liver resection. Cochrane Database Syst Rev.

[4] Pai M, Jiao LR, Khorsandi S, Canelo R, Spalding DRC, Habib NA (2008). Liver resection with bipolar radiofrequency device: Habib™ 4X. HPB.

[5] Weber JC, Navarra G, Jiao LR, Nicholls JP, Jensen SL, Habib NA (2002). New technique for liver resection using heat coagulative necrosis. Ann Surg.

[6] Taibbi A, Furlan A, Sandonato L (2012). Imaging findings of liver resection using a bipolar radiofrequency electrosurgical device - Initial observations. Eur J Radiol.

[7] Haghighi KS, Wang F, King J, Daniel S, Morris DL (2005). In-line radiofrequency ablation to minimize blood loss in hepatic parenchymal transection. Am J Surg.

[8] Gananadha S, Morris DL (2004). Novel in-line multielectrode radiofrequency ablation considerably reduces blood loss during liver resection in an animal model. ANZ J Surg.

[9] Šubrt Z, Ferko A, Jon B, Čečka F (2011). Radiofrequency-assisted liver resection: higher incidence of infectious complications?. Acta Chir Belg.

[10] Bilchik AJ, Wood TF, Allegra DP (2001). Radiofrequency ablation of unresectable hepatic malignancies: lessons learned. Oncologist.

[11] Akyildiz HY, Morris-Stiff G, Aucejo F, Fung J, Berber E (2011). Techniques of radiofrequency-assisted precoagulation in laparoscopic liver resection. Surg Endosc.

[12] Pai M, Kyriakides C, Mikhail S, Habib N, Spalding D, Jiao L, Cherqui D (2011). Radiofrequency-assisted hepatic resection. Ann Surg Oncol.

[13] Reccia I, Kumar J, Kusano T (2017). A systematic review on radiofrequency assisted laparoscopic liver resection: challenges and window to excel. Surg Oncol.

[14] Reccia I, Kumar J, Kusano T (2018). Radiofrequency-assisted liver resection: technique and results. Surg Oncol.

[15] Reccia I, Sodergren MH, Jayant K (2018). The journey of radiofrequency-assisted liver resection. Surg Oncol.

[16] Hompes D, Aerts R, Penninckx F, Topal B (2007). Laparoscopic liver resection using radiofrequency coagulation. Surg Endosc.

